# The development and pilot testing of a multicomponent health promotion intervention (SEHER) for secondary schools in Bihar, India

**DOI:** 10.1080/16549716.2017.1385284

**Published:** 2017-11-08

**Authors:** Sachin Shinde, Bernadette Pereira, Prachi Khandeparkar, Amit Sharma, George Patton, David A Ross, Helen A Weiss, Vikram Patel

**Affiliations:** ^a^ Sangath, Bardez, Goa, India; ^b^ London School of Hygiene and Tropical Medicine, UK; ^c^ Murdoch Children’s Research Institute, University of Melbourne, Australia; ^d^ Department of Maternal, Newborn, Child and Adolescent Health, World Health Organization, Geneva, Switzerland; ^e^ Centre for Chronic Conditions and Injuries, Public Health Foundation of India, New Delhi, India

**Keywords:** Adolescent, intervention development, school health promotion, India

## Abstract

**Background**: Schools can play an important role in health promotion by improving students’ health literacy, attitudes, health-related behaviours, social connection and self-efficacy. These interventions can be particularly valuable in low- and middle-income countries with low health literacy and high burden of disease. However, the existing literature provides poor guidance for the implementation of school-based interventions in low-resource settings. This paper describes the development and pilot testing of a multicomponent school-based health promotion intervention for adolescents in 75 government-run secondary schools in Bihar, India.

**Method**: The intervention was developed in three stages: evidence review of the content and delivery of effective school health interventions; formative research to contextualize the proposed content and delivery, involving intervention development workshops with experts, teachers and students and content analysis of intervention manuals; and pilot testing *in situ* to optimize its feasibility and acceptability.

**Results**: The three-stage process defined the intervention elements, refining their content and format of delivery. This intervention focused on promoting social skills among adolescents, engaging adolescents in school decision making, providing factual information, and enhancing their problem-solving skills. Specific intervention strategies were delivered at three levels (whole school, student group, and individual counselling) by either a trained teacher or a lay counsellor. The pilot study, in 50 schools, demonstrated generally good acceptability and feasibility of the intervention, though the coverage of intervention activities was lower in the teacher delivery schools due to competing teaching commitments, the participation of male students was lower than that of females, and one school dropped out because of concerns regarding the reproductive and sexual health content of the intervention.

**Conclusion**: This SEHER approach provides a framework for adolescent health promotion in secondary schools in low-resource settings. We are now using a cluster-randomized trial to evaluate its effectiveness and cost-effectiveness.

## Background

Adolescence is characterized by dramatic physical and psychological changes and alterations in social perceptions and expectations [1]. The emergence of health risk behaviours during this period has important consequences for physical and mental health, as well as for emotional well-being both during this phase of life and in later life [2]. Schools are an important platform for the delivery of health promotion interventions for adolescents, targeting risk and protective factors operating in the school environment and providing specific health-focused interventions [3,4]. Guided by the Ottawa Charter for Health Promotion (1986), the World Health Organization (WHO) introduced the concept of *Health Promoting Schools* (HPS) in 1995 [5]. This model promotes a comprehensive *total life approach* to school-based health promotion and takes into account not only the curriculum but also the school’s ethos and environment, as well as partnerships with community and access to health services [6,7]. Although the HPS provides a broad framework for action, there is little evidence that schools are able to implement this approach. In fact, few programmes have been found that implement and evaluate the HPS approach in its entirety, and most continue to use only a curriculum/social skills approach [6,7]. For many, there remains a continuing emphasis on health education rather than co-curricular health promotion [7].

India is home to around 20% (250 million) of the world’s 10- to 19-year-olds [8]. Enrolment in secondary schools has almost doubled, from 19 million students in 2000–01 to 37 million in 2013–14. This increasing enrolment offers an important opportunity for promoting health in secondary schools. Since 2005, the primary health promotion intervention in schools has been the Adolescence Education Programme (AEP) [9]. This programme is a teacher-delivered implementation of a classroom-based life-skills curriculum focused on reproductive and sexual health (RSH), and also addressing a range of other risk behaviours. Such programmes often fail to take into account the needs of particular school communities, the institutional context, and fail to value and/or measure changes in the system that may have occurred or need to occur for the sustainability of health promotion programmes [10].

This paper describes the development of the SEHER intervention (SEHER meaning 'dawn' in Hindi language; ‘Strengthening Evidence base on scHool-based intErventions for pRomoting adolescent health programme in the state of Bihar, India’), in particular its conceptual framework and delivery, and evaluates the feasibility and acceptability of this intervention through a pilot study. The overall aim of the SEHER study is to develop and evaluate a comprehensive health promotion intervention, extending the ongoing AEP (called TARANG in Bihar; TARANG means 'waves' in Hindi language) with the ultimate goal of evaluating its effectiveness against the TARANG in government-run secondary and higher secondary schools.

## Methods

The intervention development involved three stages [11]: (1) reviewing the literature on the content and delivery of school health interventions; (2) formative research to contextualize the content and delivery; and (3) pilot testing to optimize the feasibility and acceptability of the intervention. The fieldwork was carried out in the Nalanda district of Bihar, India from July 2013 to March 2015. Table 1 shows a breakdown of the outcomes from the activities conducted at each stage.

Bihar is a state in north India with a total population of over 103 million, with 22.5% of the population aged 10–19 years. Bihar was ranked 21 out of 23 major Indian states in terms of human development in 2014 [12]. Hindi is the official and most prevalent language of Bihar. Nalanda district has a population of over 2.8 million, and a literacy rate of 66% compared with India’s overall literacy rate of 74% [8]. There are 136 secondary government-run schools in the district and the Department of Education (DoE) is the main education provider. In total, 67,542 students (44% girls and 56% boys) were enrolled in these schools in the academic year 2013–14.

SEHER was implemented by Sangath, an Indian non-government organization with a long-standing record of working with adolescents, in partnership with the London School of Hygiene & Tropical Medicine (LSHTM) and the Department of Education, Bihar. All research procedures were approved by the institutional review boards of Sangath, the LSHTM and the Health Ministry Screening Committee of the Indian Council for Medical Research. Written informed consent was obtained from all the adult participants. We sought parental approval through opt-out consent for adolescent participants; in addition, assent was obtained from the adolescent participants.

### Stage 1: Reviewing the national and global literature

The goal of this stage was to identify evidence on effective school-based health promotion interventions. Systematic reviews show that school-based health promotion interventions, including HPS, bring about positive health outcomes including reduction in body mass index, increased physical activity, decreased substance use, reduced reports of being bullied, and reduced sexual risk behaviours, indicating the value of these interventions [13,14]. Of these interventions, the most effective were multicomponent, involving changes to the school environment, of long duration and high intensity, involving whole-school strategies and led by the school itself, with a school health coordinator [15]. The provision of the health promotion coordinator has been found to increase social connectedness, reduce health risks, and increase physical activity among students. However, most of the studies contributing to this evidence are from higher-income countries, mainly the USA, Europe, the UK, and Australia.

A few studies which have examined the process of planning and implementation of HPS initiatives have provided useful findings concerning the main challenges and facilitators of HPS practice. The setting-up of school-based HPS steering groups/committees provides a useful support structure for schools as they plan and design health promoting policies, strategies, and procedures [10,16–20]. These committees aim to engage with various stakeholders and work towards developing all components of a health-promoting school ethos. Perhaps unsurprisingly, this kind of shared responsibility among school staff and indeed among all stakeholders (e.g. the creation of health committees) has been identified as crucial to the success of this type of intervention [18]. At the same time, these kinds of committee can be difficult and time-consuming to develop, especially when time and resources are limited. It is also often the case that one or two champions are required to drive the initiative forward [19]. For this reason, the appointment of a health promotion coordinator to support schools in taking responsibility for the planning and implementation of health-promoting school work has been recommended [18,19]. While external guidance is clearly important, school ownership and ‘buy-in’ from all staff is also essential for a successful and sustainable initiative; again, this appears to be inextricably linked to the development of an effective health-promoting school ethos/culture [17,20].

This global evidence was supplemented by a review of case studies of four school health promotion interventions in India [21]. Drawing on information from professional networks and the academic and grey literature, 20 organisations were identified as supporting adolescent school health promotion interventions. The selected interventions were characterized by different types of human resource delivery models, different levels of engagement with the school community, and variation in scale from a pilot intervention in 10 schools to a state-wide programme in over 2000 schools. However, these case studies provide limited evidence of the impact of such interventions in influencing students’ knowledge, attitudes, and behaviours as they lacked a comparison arm. Of the four case studies, Sangath’s School HeAlth Promotion and Empowerment (SHAPE) intervention [22] demonstrated that a lay school counsellor could be an effective delivery agent, playing the role of health promotion coordinator, for a multi-component school-based health promotion intervention in Goa, which is better resourced than most Indian states. In India, school-based health promotion interventions have generally been delivered by teachers, healthcare providers, or peers who are already part of the school, as these are perceived as the least resource-intensive options. However, these interventions can compete with teaching duties and other commitments, and the sustainability of peer-delivered education interventions can be limited as the population of student peer-educators is not stable. We, therefore, decided to compare delivery of the SEHER intervention by a new, low-cost human resource (such as a lay counsellor) and an existing human resource (such as a teacher). During this stage, we also identified the intervention manuals from low- to middle-income countries that could potentially be adapted to design guidelines of intervention components in Stage 2.

### Stage 2: Formative research to adapt the intervention to the local context of Bihar

The objective of this stage was to build on the evidence gathered from the review to develop a conceptual framework, identifying the intermediate and long-term outcomes and the specific components of the interventions. Two methods were adopted: intervention development workshops with various stakeholders, and content analysis of intervention manuals for adolescent health promotion. Eight intervention development workshops – three each with secondary school students (*n* = 42), and teachers (*n* = 24), and one each with experts (*n* = 12), and project staff (*n* = 8) were conducted. Small group works and guided discussions were used to refine the content of the intervention and delivery-related issues [18,23]. Simultaneously, we reviewed the intervention manuals of 10 school-based adolescent health interventions from low- to middle-income countries [24–33] identified through the literature review during Stage 1. Data collated through these two activities was triangulated to develop a conceptual framework for the SEHER intervention and implementation strategy for each component.

## Intervention development workshops

The SHAPE programme, developed and piloted by Sangath in Goa, was chosen as the framework of the intervention as it closely matched the SEHER programme context (India), and provided evidence on effective interventions (multi-component with whole-school, group-level, and individual strategies) [22] as well as protocolized manuals for their delivery. In SHAPE, the whole-school level activities included school mapping and needs assessment; health screening camps; an anonymous letterbox for students to voice their questions and concerns (the ‘speak-out box’); a School Health Promotion and Advisory Board (comprising of headmaster, school counsellor, teachers, school management staff, and student and parent representatives) to oversee the design and implementation of the intervention in each school; and development and implementation of the school health policies. The group-level activities included classroom-based life skills training, while the individual level activities included individual counselling and referral services.

An important starting point was to describe current best practice already being implemented by the DoE. The TARANG programme comprises 16 hours of classroom sessions on life skills, including the process of growing up, establishing positive and responsible relationships, gender and sexuality, prevention of HIV/AIDS and other sexually transmitted diseases, and prevention of substance use. It is delivered by a trained teacher in each school. To avoid duplication, the classroom-based life-skills sessions were removed from the intervention, as were the health camps, which were already being organized by the DoH. The ‘Theory of Change’ to achieve our ultimate desired health outcomes emphasized that classroom sessions alone were not enough to bring change and highlighted the importance of an ‘enabling school climate’ as a key intermediary outcome. Stakeholders identified varied constructs to define school climate, including safety; quality of interpersonal relationships among students, teachers, and staff; the degree to which students, teachers, and staff contribute to decision-making at the school; feeling of school connectedness, such as responsiveness of school authorities to student concerns; school infrastructure; discipline and order in the school; quality of instructions; and dedication to student learning and achievement. Based on this theory and the recommendations from the intervention development workshops, modifications were made to the intervention. For example, we added content related to bullying and gender-related violence, rights and responsibilities, and on effective study skills, with each month of the academic year being allocated to a particular theme. As all stakeholders emphasized the engagement of peers and families, peer-groups were added to the intervention to strengthen school belongingness amongst students by providing a platform to discuss shared problems and propose solutions, and an annual workshop for parents (on ‘How to handle an adolescent’) was designed.

**Figure 1.**
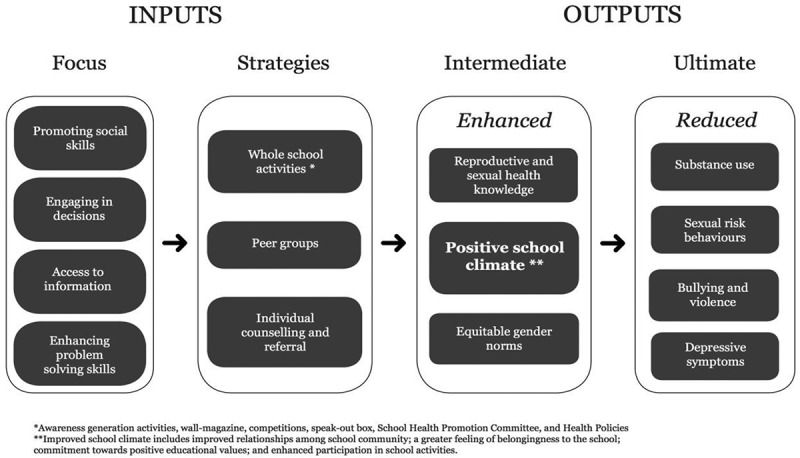
SEHER conceptual framework.

Figure 1 shows the resulting conceptual framework for the SEHER intervention, which emphasizes the importance of positive school climate. We defined this as consisting of supportive relationships among school community members, a sense of belonging to school, a participative school environment, and student commitment to academic values. The SEHER intervention identifies four priority areas for action: promoting social skills among adolescents; engaging the school community – i.e. adolescents, teachers, and parents – in school-level decision-making processes; providing access to factual knowledge to the school community; and enhancing problem-solving skills among adolescents. The strategies (whole-school, group, and individual) were largely modelled around the SHAPE intervention and comprised the following:

1. The whole-school level activities included (i) school mapping and needs assessment; (ii) activities to raise health awareness; (iii) a wall-magazine on health-related topics; (iv) extracurricular competitions; (v) an anonymous letterbox for students to voice their questions and concerns (‘speak-out box’); (vi) a School Health Promotion Committee to oversee the design and implementation of the intervention in each school; and (vii) the development and implementation of school health policies.
2. The group-level activities included peer groups of class IX students and workshops for students and teachers.
3. The individual level activities included individual counselling and referral services for students.

## Content analysis of intervention manuals

The content analysis of intervention manuals helped to identify evidence-based practices to implement the specific components in the conceptual framework, and to draft Standard Operating Protocols (SOPs). The analysis assessed, for each strategy, the adequacy of the description of its implementation, feasibility for delivery by the target human resources (see below), and the extent of adaptations needed for its use in the context. For example, the resource guide for peer leaders of the Yuva Mitr, a community-based programme to promote health and well-being of young people evaluated in a randomized controlled trial in Goa [24], provided the basis for developing guidelines of peer-group formation and facilitation. The final SOPs and resource materials were reviewed and revised for appropriateness and cultural suitability by an expert committee of the State Council Educational Research and Training, DoE, Government of Bihar, and three independent experts on adolescent health promotion in April 2014 (see Acknowledgements).

In addition to the scope and content of the intervention, the selection, training, support, and supervision requirements of the delivery agents were discussed with the stakeholders. To maintain the comparability of the two delivery formats – that is, the lay school counsellor (SEHER Mitra [friend], or SM) and the teacher (Teacher-SM, or TSM) – the stakeholders recommended keeping the curriculum the same for both but conducting the training separately to avoid contamination. The SM were required to be members of the local community, at least 21 years of age, having completed at least high school education, and with no professional health training. The TSM would be a teacher nominated by the school principal. The classroom training curriculum needed to be a maximum of six days because teachers could not provide more time due to competing commitments in the school. The classroom training was to be supplemented by a monthly group meeting for all SMs and TSMs, respectively, which served multiple roles including team-building, sustaining motivation, and shared problem-solving. On-site supervision was considered essential in order to assure the quality of the intervention and supervisors were expected to have a postgraduate degree and experience of working in the development sector. A combination of two planned and one unplanned supervisory visits per month to each school was recommended to provide support in planning and implementing activities and reviewing the intervention progress.

### Stage 3: Pilot testing of the intervention

The cluster-randomized controlled trial to evaluate the SEHER interventions selected 75 schools from a sampling frame of 112 schools (https://clinicaltrials.gov/ct2/show/NCT02484014), which were randomized to three arms (SM, TSM, and control, i.e. TARANG alone) using minimization to balance on the type of school, school size and gender composition. The pilot testing of the intervention was conducted prior to the trial (between April 2014 and March 2015) in the 25 schools randomly allocated to SM and TSM arms, respectively. We conducted the pilot in the same schools as the main trial for two reasons: (1) the secondary schools only included grade IX onwards (our primary target group for the evaluation of the effectiveness) and thus, the cohort of students to be included in the main trial would not have been exposed to the intervention during the pilot; and (2) piloting the intervention in the schools participating in the trial enabled us to embed the intervention and refine its delivery to optimize its feasibility and acceptability in each school. Twenty-nine SMs (25 in schools and four back-up SMs), 25 TSMs and eight supervisors were selected and completed their training. Each supervised both SM and TSM schools, with an average of six schools per supervisor. The 50 schools included in the pilot study had a total of 16,973 students, in Grades IX to X (8529 students in SM and 8444 in TSM schools).

We assessed the fidelity of intervention implementation (the coverage of each component, the extent to which stakeholders engaged with it and the quality of its delivery). Coverage indicators were collected through monthly reporting forms and quality indicators were assessed through ratings of specific components, such as the wall-magazine and peer group meetings, by respective supervisors and by observations made by the intervention team during field visits. At the end of the pilot, we conducted a qualitative evaluation in 12 schools (six SM and six TSM schools, respectively). The schools were purposely selected based on the high or low coverage of intervention activities. The overall aims of the qualitative evaluation were to gather multiple perspectives about the intervention components and its delivery, and to identify the gaps and improvements needed. In each school, we conducted in-depth interviews with the principal (*n* = 12), TARANG teacher (*n* = 12) and one fellow teacher (*n* = 12); and focus group discussions (FGDs) with students (*n* = 24, 12 each with boys and girls). In addition, three rounds of FGDs were conducted with the TSMs, SMs and supervisors during the intervention implementation.

The interviews and FGDs covered a range of topics including the stakeholders’ perceptions of the need for the intervention, adequacy of the content and design of supportive materials (i.e. SOPs, posters, and supplies), how school personnel and students were involved in the intervention, what support or resources were helpful for the intervention implementation, and facilitators for, and barriers to implementation of the intervention. Interviews and FGDs were conducted in Hindi and recorded. Digital recordings were transcribed and translated before coding using both *a priori* and emergent codes by two researchers (S.S. and B.P.) using NVivo version 10 (QSR International, Burlington, MA, USA). Data collection and analysis progressed iteratively, identifying and interpreting themes, leading to modifications to the intervention. A thematic analysis was used for data analysis, with codes and qualitative results discussed by the team to achieve consensus. The following analytical themes and subthemes were employed for the synthesis and interpretation of the quantitative and qualitative data:

- Acceptability: Is the need for the intervention acknowledged by the stakeholders; stakeholders’ acceptability of the intervention content; and stakeholders’ participation in and attitudes towards the intervention activities.
- Feasibility: What was the implementation of the intervention activities compared with what was planned; stakeholders’ perceptions of the barriers to, and facilitators of, the introduction and delivery of the intervention activities; systems for implementation and supervision.

## Results

### Acceptability

The school principals, teachers, TSMs, and SMs were almost unanimously of the view that the intervention was meeting an important need, considering the fact that students in government schools are socially and economically disadvantaged and require health and social skills training. Drawing attention to the low status of women in the region, many stakeholders also spoke of the potential of the intervention to raise awareness of reproductive and sexual health issues, gender equity, and gender-based violence to change the outlook of youth.

What is special about this programme is that the grade IX and X students are getting health education through participatory methods. There is no teaching as such, but the whole school is engaged in delivering key messages like what is gender-based violence, what are key reproductive and sexual health issues, what is anxiety and stress, how to handle stress and so on. (Principal of a SM school)

Of all the stakeholders interviewed, only one school principal and three teachers expressed lingering doubts about the desirability of providing information on sex and pregnancy to adolescents. They feared that this might have negative consequences, such as heightening attraction to the opposite sex, which, in turn, could result in a growing number of romantic and/or sexual relationships.

One thing I don’t like about this programme is that the kind of information given to these children on adolescence … these children are not mature enough to understand these things; they may act on the information, which is not good. (Teacher of a TSM school)

The school principals, teachers, SMs, and TSMs acknowledged that through the meetings with the staff and SHPC members, the schools identified issues of concern and reviewed the practices and priorities of their schools. For many schools, the discussions during these meetings provided the impetus for shared planning and action. For example, in two schools, the SHPC meeting discussed the need for separate toilets for boys and girls and networked with the District Education Office for financial support. As a result, these schools received funds to build toilets.

The school staff also acknowledged that a lot of changes took place in the school due to the programme. For example, teachers mentioned that a regular whole-school assembly was being organized, and the chits submitted by the students in the speak-out box motivated the teachers to follow a daily school schedule. Teachers mentioned that through activities like whole-school assembly sessions, wall-magazine development and competitions, the interactions between students and teachers improved significantly.

Apart from providing information to the students, SEHER has been useful in bringing positive changes in our school. Earlier, there was no student assembly organized in our school;however, since joining, the SEHER-Mitra is facilitating the daily assembly. Students are participating in the various activities during the assembly like newspaper reading, cleanliness drive, skit presentation, and so on. Similarly, the daily schedule was not being followed in our school, but a large number of students have demanded a regular schedule of classes in the school through chits, which has pressurized the headmaster and teachers to follow a schedule. (Female teacher from SM school)

Although the SMs and TSMs were generally enthusiastic about the intervention, two of each, all from lower-performing schools, mentioned that support from fellow teachers was not always forthcoming, either because they did not consider the subject important or because they did not approve of the content of the intervention. Several teachers complained that not enough information was shared about the intervention.

Students were unanimous across both intervention arms in their enjoyment of the activities, and equally unequivocal that the activities and topics were interesting and informative. Specifically, students praised the activities like debates and panel discussions, storytelling, and roleplays conducted during the whole-school assembly; the development and display of a monthly wall-magazine on various topics; and organization of monthly competitions. Students also appreciated the provision of the speak-out box, as they could submit their concerns and complaints. However, several students in the TSM arm said that they were apprehensive about sharing their complaints through the speak-out box because they were not sure how the TSM would react. The boys from both arms suggested including activities that would increase their participation in, and engagement with, the intervention such as having more competitions and physical activities. Overall, the students participating in the FGDs described the SEHER intervention as ‘very useful’ and ‘helpful for our future careers’.

Students like us, living in villages, face multiple problems. Many students drop out due to the poor financial status of the family. Girls are forced to marry before they complete their education or are asked to sit at home to finance the education of the male child, and so on. Many girls do not come to school during ‘those’ days [referring to menstruation] because there is no separate toilet for girls in the school. Our science teachers do not talk about these topics in the classroom and ask us to read about it on our own. Now we can directly go to the TSM or drop a chit in the box and ask our questions, and seek guidance. I myself have sought his advice for my difficulties and it has helped me. (Female FGD participant from TSM school)

In general, the students’ perception of the SM was favourable and they perceived the SM as someone who was readily available in the school, was ‘friendly’ and was more approachable than other teachers, and because s/he could help them in solving their issues while maintaining the confidentiality. The TSMs were also perceived favourably, although some students mentioned that they were not always available and/or approachable due to their other engagements in or out of the school. Some students were not confident that the TSM would respect confidentiality and share their issues with other teachers. In addition, the TSMs often felt overloaded due to other competing assignments such as teaching of regular syllabus, administrative tasks, election duties, answer sheet evaluations, etc. Their motivation was based only on their enthusiasm; they did not get any additional pay or benefits for this work.

### Feasibility

#### Intervention coverage

At the beginning of the academic year, the intervention team set detailed targets for intervention activities. Coverage of the monthly intervention components was generally high in both arms (Table 2) with the exception of awareness-generation activities during the school assembly. This was not organized daily in many schools until the SM/TSM were able to persuade the headmaster to start this practice. The acceptability of the intervention during the latter six months (September 2014 to February 2015), was reflected by the high number of submissions to the speak-out box and the number of students who have availed counselling services. The coverage of some intervention activities was higher in SM than TSM schools. For example, the SM schools conducted more whole-school assembly activities (75% versus 41%) and staff meetings (96% versus 68%) than TSM schools. Similarly, the SM schools received more chits through the speak-out box (527 versus 321) and more students accessed counselling services in these schools than the TSM schools (2.38% versus 1.80% of the total student population). These quantitative indicators were consistent with qualitative data and helped in identifying facilitators of, and challenges to, intervention delivery.

**Table 1.** Results from application of three-stage methods for development and testing of SEHER intervention.

Stage	Objectives	Outcomes
Evidence synthesis	Identify evidence on effective school-based health promotion interventions	School-based health promotion interventions have produced small to moderate effect sizes on range of adolescent health outcomes including: physical activity; tobacco, alcohol and drug use; bullying; sexual risk behaviours; and mental health Multi-component and whole-school interventions, delivered within a supportive school environment, show more potential for health promotion than only classroom-based curricula Provision of a Health Promotion Coordinator has been found to increase social connectedness, reduce health risks, and increase physical activity among students A school-based steering committee is essential for planning and designing health policies and activities
(2) Formative research		
2A. Intervention development workshops	Develop a conceptual framework of the intervention Identify the intermediary and long-term outcomes Identify the specific components of the interventions Identify the selection, training, and supervision requirements of the delivery agents	**Content of the intervention** Focus of the intervention on building ‘school climate’ as the primary intermediary outcome Identified following long-term outcomes: reduction in substance use, sexual risk behaviours, bullying and violence, and depressive symptoms Identified four priority areas or foci for action: (i) promoting social skills among adolescents, (ii) engaging the school community in the school-level decision making processes, (iii) providing access to factual knowledge to the school community, and (iv) enhancing problem-solving skills among adolescents Intervention activities organized at three levels: whole-school, group, and individual Avoid duplication of existing intervention elements, and thus drop classroom-based life skills sessions (already being delivered by the TARANG programme in the study context) Added peer groups to intervention component to strengthen school-belongingness among students **Selection, training and supervision of the delivery agents** Two human resources identified for the role of health promotion coordinator: a new, low-cost human resource (the lay counsellor) and an existing human resource (a teacher) Selection criteria for lay counsellors: to be members of the local community, ≥21 years of age, have completed at least high school education, and have no professional health training Selection criteria for teachers: have a ≥5 years’ experience of teaching in secondary schools, have ≥12 years of service remaining, not teaching TARANG curriculum, and willing to undergo a weeklong residential training Six-day training curriculum to train lay counsellors and teachers; supplemented by monthly group meetings for lay counsellors and teachers Separate training and monthly group meetings to avoid contamination A combination of two planned and one unplanned supervisory school visits per month to each intervention school
2B. Content analysis of intervention manuals of adolescent health promotion	Identify evidence-based practices to implement the specific components laid out in the conceptual framework, and draft the standard operating protocols for each component	Adapted and defined standard operating guidelines for intervention components based on evidence-based practices to local context
(3) Pilot testing	Evaluate the acceptability and feasibility of the intervention, and identify the gaps and improvements needed	**Acceptability** Intervention activities were well accepted in most schools Perceived as meeting important needs of students Acceptance of both types of delivery agents One school dropped out as the school management perceived the reproductive and sexual health-related content inappropriate **Feasibility** High coverage of whole-school activities in both arms; higher intervention coverage in the lay counsellor arm relative to the teacher delivery arm Key facilitators: participatory nature of intervention activities, availability of platforms to raise students’ concerns, redressal of students’ complaints or problems while maintaining confidentiality, engagement and support of the headmaster, and involvement of other teachers in intervention activities Key barriers: lack of engagement of teachers in intervention activities, lack of male students’ participation, fall in students’ attendance in last months of the school year (after January), and non-availability of teachers during ‘unplanned’ supervisory visit due to their competing teaching responsibilities **Strategies employed to address barriers** Added teachers’ monthly meeting as a component to improve teachers’ engagement in intervention activities Added monthly intra-school competitions to increase boys’ participation Focus on completing all core intervention components between June and January Monthly unplanned supervisory visit per month changed to a planned visit to lend more support to teachers and lay counsellors

**Table 2.** Coverage of SEHER activities in pilot study (September 2014 to February 2015).

	School level target	TSM arm (*n* = 25)	Coverage	SM arm (*n* = 25)	Coverage
Awareness generation					
Number of assemblies addressed	4/month	246	41%	450	75%
Number of staff meetings	1/month	102	68%	144	96%
Wall-magazine					
Number of issues	1/month	135	90%	144	96%
Speak-out box					
Number of chits received	–	321	–	527	–
Number of chits addressed	–	230	–	353	–
Number of chits not addressed	–	91	–	174	–
School Health Promotion Committee					
Number of meetings	1/year	25	100%	25	100%
Workshops					
Number of workshops with students	1/year	25	100%	25	100%
Number of workshops with teachers	1/year	25	100%	25	100%
Individual counselling					
Number of cases (total number of students)	–	152 (8444)	–	203 (8529)	–
% of students who accessed counselling	–	1.80	–	2.38	–

#### Facilitators to intervention delivery

Students described the participatory nature of the activities, availability of platforms such as the speak-out box and the peer groups to raise their concerns, recognition of contributors to the wall-magazine or daily assembly, redressal of students’ complaints or problems while maintaining the confidentiality as the facilitators to the engagement with intervention components. Other key factors in the high-performing schools were the engagement and support of the headmaster, ownership and participation of other teachers, and ongoing training and continuous professional support through supervisory and, once each month, day-long meetings for the SMs and TSMs. In the TSM schools, the monthly ‘unplanned’ supervisory visit was not serving its purpose as the TSMs were often unavailable due to their competing teaching responsibilities. Consequently, the monthly unplanned supervisory visit per month was changed to a planned visit to lend more support to the TSMs in completing their targeted activities. The SMs and TSMs mentioned that the monthly group meetings increased their confidence in implementing SEHER activities, provided a platform to discuss school-level challenges and generate solutions, and served to motivate them. The supervisors mentioned that intense monitoring and supervision of the intervention was one of the factors that helped in quickly identifying bottlenecks in the delivery and addressing these.

#### Barriers to whole-school level activities

Common barriers to the delivery of the whole-school level activities included lack of engagement of teachers in SEHER activities, lack of boys’ participation, negligible participation of parents in school governance and day-to-day proceedings, and a fall in students’ attendance after mid-December/early January once government incentives for attendance were disbursed. The lack of teacher engagement was addressed through a regular monthly meeting with the school staff to review the previous month’s activities and plan for the upcoming month. In addition, all the teachers, instead of a few representatives, were invited to be a part of the School Health Promotion Committee. This increased the engagement of the headmaster and teachers, and moved the ownership of the intervention to the teacher community. Male student involvement was improved through expanding the scope of the peer-group and wall-magazine activities through school-wide monthly competitions (e.g. on elocution and drawing), and increasing the number of peer groups from one for the entire grade to one for each division of the grade (on average, there were two to four classes of grade IX in each school). The challenge of the fall in attendance led to increased focus on completing all core intervention components between June and January. One barrier that the intervention was unable to address was the low participation of parents, reflected in the very low attendance in the annual workshop on ‘handling adolescents’, despite multiple efforts including letters, phone calls, and personal visits; this resulted in dropping the annual workshop with parents from the intervention.

The barriers to the delivery of whole-school activities were different in the SM or TSM schools. One of the TSM schools opted out after completing the pilot study as the school management committee perceived the reproductive sexual health content of the intervention unacceptable for the secondary students. In three SM schools, headmasters and senior teachers felt that the SMs were not equipped to facilitate such an intervention as they were younger than the teachers in the school, did not have appropriate educational qualifications or experience to work in the school setting, and were perceived as external to the school. This feeling was stronger in those schools where the SMs shared with the headmasters that students complained about the disciplinary practices of some teachers. This challenge was addressed by including an annual workshop for the teachers on ‘discipline practices’ and through engagement with all teachers in the intervention, as described earlier. This resulted in reducing the performance anxiety of the SMs and improving other teachers’ participation and support in the intervention activities.

#### Barriers to group-level activities

The barriers observed in organizing peer group activities included the responsible peer group member not fulfilling their duties, unclear understanding of the group’s objectives and role, hesitance to participate in mixed-gender group activities, and irregular attendance at the group meetings. These barriers were addressed through highlighting the importance and role of such groups to the members in the monthly meetings. The participation of group members increased gradually due to enhanced diversity and frequency of the intervention activities – such as celebration of important days related to health, organization of monthly competitions, and a school-level cleanliness drive.

#### Barriers to individual-level activities

In most schools, girls were unwilling to consult male SM/TSMs for counselling services. As it was not feasible to provide SM/TSMs of both genders in each school, female students were encouraged to contact a female teacher in the school to discuss the sensitive issues if they did not wish to consult the male SM/TSM. Through the awareness-generation meetings during the daily assembly, the SM/TSMs assured students of confidentiality and wherever possible made a private space available for a confidential counselling session. A key barrier observed in TSM schools was the TSM’s inability to switch swiftly between the role of a teacher and counsellor; some students felt uncomfortable or uncertain about seeking counselling from the TSM on matters that may be perceived as sensitive or concerning the school environment.

## Discussion

The evidence base for the design and sustainable delivery of school health promotion interventions from low- and middle-income countries is limited. This paper describes the development and piloting of the SEHER intervention in the state of Bihar, one of the most socio-economically disadvantaged states of India. Our goal was to design a health promotion intervention that extended the ongoing classroom based life skills programme (called TARANG in Bihar) with the ultimate objective of evaluating its effectiveness against the TARANG in government-run secondary schools. Our methodology was aligned with the approach recommended by the MRC framework for the design and evaluation of complex interventions [11] and involved three stages of evidence synthesis, formative research, and a pilot study.

The existing literature, the vast majority of which is from high-income countries, shows that whole-school and multicomponent interventions have more potential for health promotion than purely classroom-based curricula and that the provision of a health promotion coordinator increases social connectedness, reduces health risks, and increases physical activity among students [16,18,19,34]. Our study builds on this evidence in several ways: first, the SEHER intervention emphasizes the mediating role of school climate in improving health and health-related outcomes among adolescents; second, we demonstrate that a range of participatory delivery methods – including daily school assembly-based activities, within-school competitions, wall-magazine, speak-out box, and school health promotion committee – enhances the acceptability of the intervention; third, the role of the health promotion coordinator can be played by either existing teachers or lay counsellors (low-cost additional human resource); fourth, better coverage and acceptability of some intervention activities (e.g. weekly assembly activities and counselling) are observed in the lay counsellor arm than the teacher arm, but as the latter involves less cost, a comparison of these delivery arms on outcomes and costs is required to inform policymakers of the relative advantages and limitations of each delivery model; and finally, the participatory nature of intervention activities, availability of platforms to raise students’ concerns, redressal of students’ complaints while maintaining confidentiality, engagement and support of the headmaster, and involvement of other teachers in intervention activities are key requirements for acceptability and feasibility.

A key outcome of this process was a recognition of the complex pathways through which such multicomponent interventions exert their ultimate effects on health outcomes and the crucial role of intermediary outcomes, notably school climate, in this pathway (Figure 1). Thus, the stages of the intervention development moved the theory of the intervention from directly influencing the student health outcomes to engaging the school community in building the school climate, which then plays a mediating role in facilitating positive health outcomes. Identification of these pathways also influenced the choice of primary outcomes within the relatively short period available for a definitive randomized controlled trial. The intervention framework extended the narrow view of school health as primarily focused on classroom curriculum or health education to the broad, multiple strategies of policy, improving the school’s physical and psychosocial environment and providing a range of services – all with participation of headmaster, teachers, and students, and coordinated by a health promotion coordinator in the form of a teacher or lay counsellor. These findings are consistent with the evidence which shows that the participation of teachers, family, community members, and students in the design and implementation of the programme, and the use of participatory, active learning techniques where young people have the opportunity to practice or demonstrate specific skills, are key drivers of the acceptability and feasibility of a school health promotion intervention [7]. Similarly, individuals who strongly support and advocate for a programme have often been cited by the education and health agencies as among the primary reasons they have been able to implement innovative programmes [35–38]; in our study, the leadership of school principals was a key factor in the successful implementation of the intervention and facilitating the engagement of the wider school community.

## Conclusions

The relationship between school enrolment, participation, and improved health outcomes is well established [7]. With rapid extensions to education in many developing countries, including in South Asia [39], secondary schools have the potential to be an important platform for health promotion and prevention. However, most evidence about the effectiveness of multicomponent HPS interventions for behaviour changes is from developed countries or settings in middle-income countries where schools are relatively better resourced, with relatively high levels of literacy and social inclusion [8,13–15,30]. The SEHER intervention builds on this evidence and was designed for the most poorly resourced educational sector in a population with very low human development indices. Following a systematic methodology, we have attained our goal of developing an intervention that is acceptable to various stakeholders, feasible to deliver, is designed to be scalable, and has a clear Theory of Change in which influencing school climate lies at the heart of achieving desirable long-term behavioural and health outcomes. The SEHER intervention development process also largely confirmed the acceptability and feasibility of the components of the intervention as originally developed and piloted in the relatively more advantaged state of Goa to the Bihar context, indicating their generalizability to the widely varying socio-cultural settings of schools in India. The resulting intervention, delivered either by a teacher or a lay school counsellor, is now being evaluated in a cluster randomized controlled trial (NCT02484014). If either delivery arm is found effective and cost-effective, it has the potential to be a model for promoting health and well-being in other low-resource school settings.

## Paper context

In this paper, we report on the development and piloting of a multicomponent school-based health promotion intervention in secondary schools in Bihar, India. Evidence from systematic reviews, formative research, and a pilot test in 50 schools led to the design of the SEHER intervention, which focused on promoting school climate to build social skills among adolescents, engage adolescents in school decision making, provide factual information on health and its determinants, and enhance problem-solving skills. Specific intervention strategies were delivered at three levels (whole school, student group, and individual counselling) and coordinated by either a trained teacher or a lay counsellor. The intervention showed generally good acceptability and feasibility of the intervention. We are evaluating the effectiveness and cost-effectiveness of these two delivery models in a cluster-randomised trial in Bihar.
